# Knee Orthotics Do Not Influence Coordinative Skills—A Randomized Controlled Crossover Pilot Trial

**DOI:** 10.3390/jpm12091509

**Published:** 2022-09-14

**Authors:** Robert Prill, Caren Cruysen, Aleksandra Królikowska, Sebastian Kopf, Roland Becker

**Affiliations:** 1 Center of Orthopaedics and Traumatology, University Hospital Brandenburg/Havel, Brandenburg Medical School Theodor Fontane, 14770 Brandenburg an der Havel, Germany; 2 Faculty of Health Sciences Brandenburg, Brandenburg Medical School Theodor Fontane, 14770 Brandenburg an der Havel, Germany; 3 Ergonomics and Biomedical Monitoring Laboratory, Department of Physiotherapy, Faculty of Health Sciences, Wroclaw Medical University, 50-367 Wroclaw, Poland

**Keywords:** anterior cruciate ligament, biomedical monitoring, physiotherapy, rehabilitation, return-to-sports, sports

## Abstract

Objective: This single-blind randomized controlled crossover pilot trial investigated whether hard or soft knee orthotics affect the back in action (BIA) test battery performance. Methods: Twenty-four healthy participants (13 males, 11 females) were randomly assigned into three equal groups differentiated through the order of device use. The data were collected in a laboratory setting. BIA test battery (balance tests, vertical jumps, and parkour hop tests) was run with a rigid orthotic device, a soft brace, or no aid in a crossover order. Analysis of Variance repeated measures and Friedman Test were used to calculate depended-group differences. Results: No significant or clinically relevant effect or differences was observed between running the BIA with a soft brace, rigid orthosis, or no aid (*p* = 0.53–0.97) for all included tests. No adverse events have been observed. Conclusion: Soft and rigid knee braces do not affect performance in healthy participants. Missing experience with the devices might explain a few influences on feedback mechanisms. There is no disadvantage to be expected regarding healthy participants running back to sports.

## 1. Introduction

According to the International Organization for Standardization (ISO), orthoses, called an orthotic device, is defined as an externally applied device used to compensate for impairments of the structure and function of the neuro-muscular and skeletal systems [1]. Consecutively, knee braces have been classified by the American Academy of Orthopedic Surgeons into prophylactic, functional, rehabilitation, and patellofemoral ones [2]. Even though they are widely used, controversy exists about whether braces should be used for protection or treatment purposes, especially for the knee during and after rehabilitation. For instance, braces and orthoses are well-established for rehabilitation after anterior cruciate ligament (ACL) injury, but their usefulness is still discussed with controversy [3]. Prophylactic knee bracing does not seem to reduce the incidence of knee injuries in uninjured patients [4].

There is also conflicting evidence in terms of the impact of braces on knee proprioception. It has been shown that orthoses did not seem to increase kinesthetic awareness [5]. The role of proprioception in motor control and functional joint stability seems to play an important role. More attention should be paid to the role of feedforward and feedback strategies [6].

Another common question when returning to sports is if braces or orthoses may impact athletes’ performance. A review comparing functional bracing with non-bracing after Anterior Cruciate Ligament Reconstruction (ACLR) showed significant improvement in knee kinematics and gait, but decreased quadriceps activation without affecting function tests, range of motion, and proprioception [3]. However, reducing quadriceps activation may cause the impact of specific tests such as hop tests.

The purpose of this crossover trial was to evaluate the impact of hard and soft orthoses on the performance of healthy subjects. It was hypothesized that braces or orthosis might reduce performance when completing the back in the action (BIA) test battery. To prove this hypothesis, we developed a crossover design with healthy adults, running a test battery without any knee stabilizers aids, a brace, and an orthosis.

## 2. Materials and Methods

A single-blind controlled crossover design was used and the study was conducted in accordance with CONSORT checklist of information to include when reporting randomized crossover trials [7]. Current recommendations for reporting sports medicine and orthopedic clinical trials have been respected [8]. The study was approved by the appropriate ethical committee related to the institution in which it was performed. All the participants were voluntarily recruited from medical and physical therapy students. Inclusion criteria were the age of 20 to 30 years, healthy well-being, moderate sports activity, and the absence of current pain in the limbs or trunk. Subjects with a previous trunk, hip, knee, or ankle surgery were excluded. Subjects were selected by the person who also instructed the BIA test battery. This person was not involved in the statistical analysis. After written informed consent was obtained, subjects were randomly allocated to run 1, 2, or 3 using the Microsoft Excel (© 2022 Microsoft Corporation) function with the formula ‘= INT(RAND()*(4 − 1) + 1)’ by a not further involved person, resulting in a random allocation of 1:1:1 for all three types of runs. They either started with “soft brace,” “hard Orthotic device,” or “no aid,” followed by the other two in a different order (Table 1).

**Figure 1:**
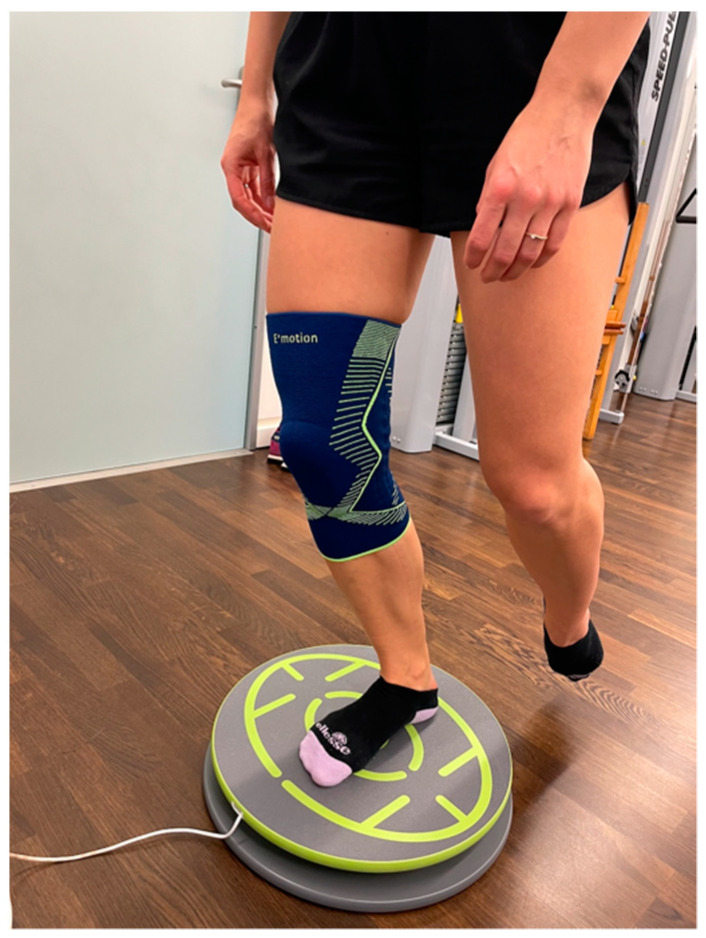
Single Leg Balance Test.

**Figure 2:**
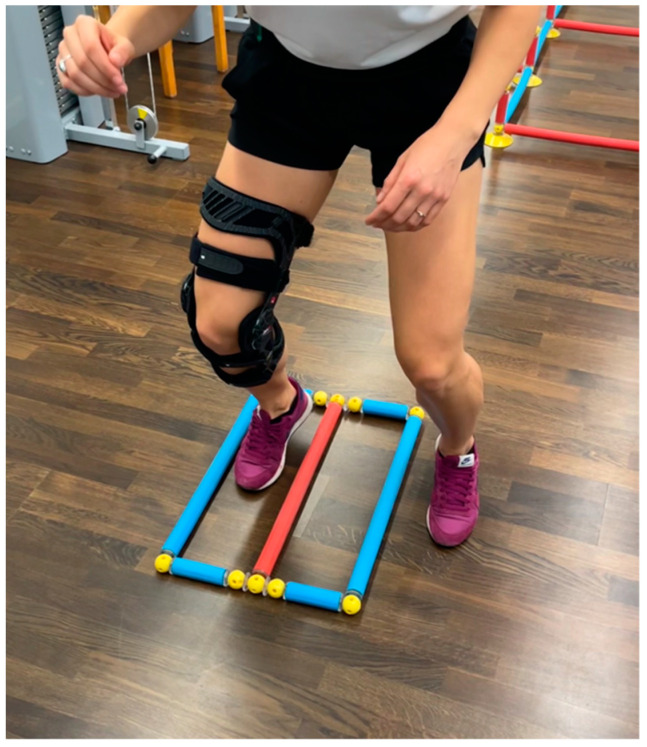
Quick feet test.

All three groups, being actually differentiated through the order of used devices they perform the test battery with, ran the described parkour three times with a full rest of about 40 min in between: once without an external stabilizer, once with a functional brace (*Genumedi^®^ E+motion*, *Medi*, *Bayreuth*, *Germany*) as presented in Figure 1, and once with an orthosis (*M.4s^®^ comfort*, *Medi*, *Bayreuth*, *Germany*) as presented in Figure 2.

Randomizing was essential to reduce the interaction effects of the three runs of the test battery. Participant blinding was not possible due to the nature of the study.

A simple power-based effect size calculation to detect a large effect of not wearing external knee stabilizers was performed for three groups using G*Power 3.1 (Duesseldorf, Germany). with an alpha error of five percent and a power 1-β of 90 percent, suggesting a sample size of 24 to be included.

**Figure 3:**
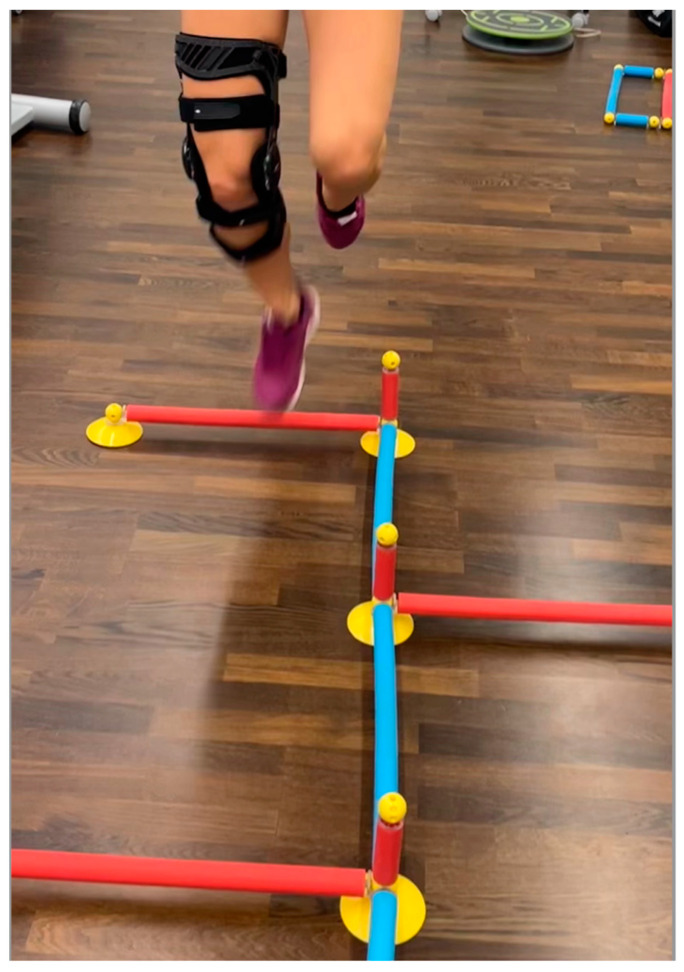
Speedy Jump Test.

The BIA consists of stability tests, two-leg and one-leg countermovement jumps (CMJ), standardized hop parkour, and a speed test [9]. The BIA showed excellent test-retest reliability for two-leg CMJ, moderate reliability for the two-leg stability test, and good reliability for all further included test items [10]. Individuals did warm up with a standardized protocol, including 10 min (min) of ergometer cycling and one test run through all tests of BIA. The BIA test battery was already described in detail [9,10]. In brief, it includes seven tests. First, individuals must balance with both legs for 30 s (s) on a balance board (*TST Trendsport*, *Grosshöflein*, *Austria*), measuring the center of pressure and giving instant biofeedback to participants about the position of the disc (Figure 1). A higher score will be reached when few movements and centered balance are measured. The second task is similar, but patients stand on one leg only, first on the dominant and second on the non-dominant leg. The third task is a two-leg countermovement jump (CMJ) wearing a movement sensor (*Myotest S.A.*, *Sion*, *Switzerland*) in a belt for capturing height differences. A one-leg CMJ follows a two-leg CMJ, performed in the same manner just with a single leg. This is followed by a three-repetition plyometric jump test. The sixth task is a speed jump test. Hereby, a standardized single-leg hop parkour test (*TST Trendsport*, *Grosshöflein*, *Austria*) (Figure 3) with 16 forward-backward and sideway jumps must be completed with both dominant and dominant non-dominant legs.

The seventh test is a quick feet test using the Speedy Basic Jump Set (*TST Trendsport*, *Grosshöflein*, *Austria*, *Figure 2*). Time for 15 repetitions was measured. When unexpected events occurred, like participants stepping down from the challenge disc or jumping in the wrong order, they were asked to start the attempt again. The tests were performed according to a previously published quality criteria study [10]. After the 24 participants ran the test, battery results were blinded and transferred to an examiner. Without the knowledge of group assignment, an initial statistical analysis was performed.

IBM SPSS Statistics V28.0.0.0 was used for statistical calculations. Descriptive statistics were used to show data distribution. Data were presented as median and interquartile ranges. Analysis of Variance repeated measures (ANOVA) and, due to non parametric data in the jump test being ordinal scaled, the Friedman Test was used to calculate dependend between-group differences. For additional blinding, a group assignment was added to the results after statistical calculation and discussion of results in the author team.

## 3. Results

**Figure 4:**
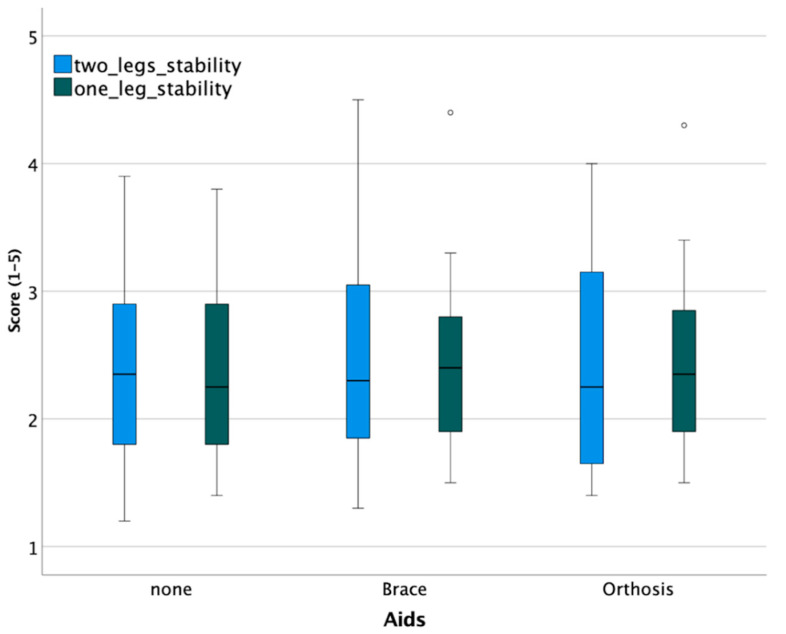
Stability tests show no clinically relevant difference between devices (° are outliers).

Twenty-four subjects (13 female, 11 male) with an age of 24 ± 2 years, a height of 1.75 ± 0.08 m, and a weight of 65 ± 8.5 kg were included. The median score for the two-leg stability test was 2.3 (1.72 to 2.90,) and for the one-leg stability test, 2.3 (1.90 to 2.88) (Figure 4).

A statistically significant difference was not observed between groups (Table 2).

**Figure 5:**
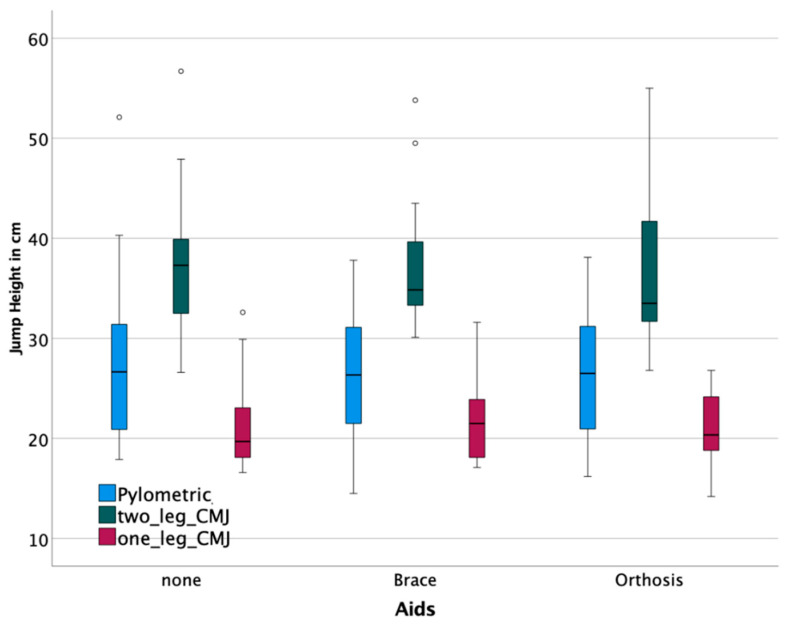
Jump tests show no clinically relevant difference between devices. (° are outliers).

Participants performed the plyometric jump test without any knee stabilizer with a mean jump height of 27.6 ± 8.4 cm, with a brace of 25.9 ± 7.0 cm, and with an orthosis of 25.9 ± 6.4. For two-leg CMJ, the range of means between the three groups was 36.6 to 37.3 cm and 21.0 to 21.7 cm for one-leg CMJ. Median and IQRs are displayed in Figure 5.

None of the between-group mean differences were significant (Table 2). Subjects completed the parkour on average in 7.8 ± 3.6 s, with a group range of means between 7.68–7.88 s. The quick feet test was finished with a mean time of 7.68 ± 1.06 s without aids, 7.72 ± 1.58 s with a brace, and 7.88 ± 1.15 s with an orthosis. None of the between-group mean differences were significant (Table 2).

## 4. Discussion

Our main findings in the present study were that no significant differences in performance of the back in action test battery were observed, regardless of if participants performed the test battery with a hard orthotic device, a soft brace, or no aid. Therefore, those devices did show a significant or clinical relevant effect on the performance of the participants.

The BIA consists of stability tests, two-leg and one-leg CMJ, standardized hop parkour, and a speed test. The BIA showed excellent test–retest reliability for two-leg CMJ, moderate reliability for the two-leg stability test, and good reliability for all further included test items [9]. The current test battery was used to test more than one skill and allow a better overview of coordinative abilities and the possibility to draw conclusions on the physiology.

Initiation of feedback control is based on actions predominantly influenced by the experience with the detected stimulus, which could be somatosensory, visual, or vestibular. The answer of the organism is based on different regulatory systems like reflexes or experience-based modulation. In contrast, feedforward control is based on anticipatory actions before homeostatic disruption is detected with one of the before-mentioned stimuli and is immediately used until feedback mechanisms help better react to a specific task [11,12]. Again, feedforward control is based on experiences with the expected task.

Feedforward strategies are predominantly used when motor learning for a certain task is finished and a person is familiar with the task. When external factors are likely to influence the success during the process of answering a task, like in Judo, feedback mechanisms become more important [13]. The functional joint stability is accomplished through a complementary relationship between static and dynamic components [12].

A potential explanation for similar results with or without orthotic devices might be few kinesthetic benefits for healthy participants due to the rather fixed and feedforward-based neuromuscular motor program they use for answering the tasks. One potential explanation is that lacking the experience for benefiting from feedback advantages, they could take additional external proprioceptive information through braces or orthosis.

For example, the combination of laterally wedged insoles and knee braces might influence balance in medial knee-osteoarthritis patients [14]. In contrast to those findings in our study, no influence was observed on a single leg or two leg balance tests performance, which is predominantly feedback control-based, which could be explained through the few benefits participants can take out of the additional sensory information. There was no clinically relevant difference between groups observed in the Counter Movement Jump Tests, parkour, and quick feed tests. This is an indicator of not being handicapped in developing speed and power by the used orthotic devices.

The same test design might show different results in patients after ACLR. The feedforward and the feedback systems are likely to be negatively influenced through not properly working active contributors, as to be seen in the onset latency of muscle activation. Neuromuscular impairments are present in patients early and late after ACLR [15,16]. The Sensorimotor system is also not sufficiently working through the absence of passive contributors like the ACL and misinforming structures like the swollen and inflamed joint capsular after surgery [17]. This results in high-risk knee positions. For example, larger knee abduction during landing seems to have a predictive value for ACL ruptures [18]. There probably is a mechanical, passive function of braces, as some impact of braces on physical performance was shown. The usage of braces or sleeves reduced the maximal flexion angle, abduction angle, and adduction moment of the knee [19]. Correct aligned knee brace might also slightly reduce ligament strain [20]. Therefore, feedforward and feedback strategies are likely to be negatively influenced by injury and reconstruction of the ACL, and those systems might benefit from braces or orthosis if athletes gather a certain amount of experience. This means that the longer the brace was used, the more benefits the participant could perhaps take out for more advantages in terms of feedback.

Strategies for test batteries are strongly discussed, and approaches like indexes, sensor-based testing, or rehabilitation strategies become more popular [21,22]. While significantly higher values in healthy athletes and patients after ACLR performing a drop jump with a knee brace [23] or for symmetry in increased hop distance during the single and crossover hop tests have been observed [24], no differences in performance for patients after ACLR in jump height or a modified agility t test have been seen at the timepoint of return to sport (RTS) and three months after [25]. In an RCT, including 150 patients post-ACLR, no significant difference was observed in the short or long term for the outcomes ACL-QOL Questionnaire, KT-1000 arthrometer side-to-side difference, for hop limb symmetry index, or Tegner Activity Scale between patients wearing neoprene sleeve or hard orthotic device. In a study with 19 male alpine skiers, neither rigid knee braces nor a sleeve influenced negative drop jumps. A musculoskeletal modeling analysis showed reduced flexion and frontal plane movement when wearing one of the devices, but they did not help reduce any shear force in the knee [19].

With respect to these controversies, the usage of hard or soft orthotic knee braces generally seems to result in some benefits with regard to of safety, but not in disadvantages in terms of performance.

In a recent study, athletes with ACLR eleven months after surgery were evaluated. They still showed a side-to-side difference (LSI) in the BIA test items. This was in contrast to the four-hop test and the measured isokinetic strength, which both did not show side-to-side differences anymore. This indicates the test might be superior compared to a single hop or isokinetic test for detecting limb symmetry index (LSI) differences after surgery, and thus is a better guide to allowing an athlete [9]. Using the BIA test battery, it was shown that patients eight months after ACLR were not ready for a safe RTS in terms of neuromuscular abilities [26]. These findings align with another study showing that nearly half of highly active patients present strength and functional fitness deficits six months after ACLR, no matter which graft type was used [27]. Other factors such as the sensorimotor system may be considered at the time of return to sports. Even athletes with higher strength and function results were at similar risk of an ACL graft tear with a higher risk for a contralateral ACL tear. High-performance patients are also at increased risk for an ACL graft tear [28]. Therefore, thinking about evaluating expressions of the Sensorimotor System must not only be done in terms of the system; it must be done in terms of the demands as well. Kyritsis et al. showed predictive value for isokinetic strength testing, a running *t*-test, triple-hop, and triple crossover hop test, and also found a decreased hamstrings to quadriceps ratio of the involved leg to be of higher risk for graft rupture [29]. Besides the additional use of established questionnaires, like the Lysholm Score [30] and generally the inclusion of psychological factors for RTS decision making [31], new issues should be considered when carrying on defining back to sport criteria. Activities around the hip joint, gluteal muscles, and lateral trunk obliquity seem to be of high interest for evaluating the RTS performance [32,33,34]. Additionally, fatigue should be more highlighted in ACLR risk evaluations [35]. More activity of the hamstrings seems to be promising for less ACL loading and, therefore, less risk of re-rupture [36]. This should be considered for improving rehabilitation programs, perhaps brace designing, and RTS battery development.

The following limitations of the study should be mentioned. Despite the possibility of a few feedback advantages, the results of the balance tests show a widespread distribution of performance. Therefore, the measurement error might be larger than the influence of orthotic devices on performance due to a lack of discriminatory power of the test in the studied population. The used test battery was developed for athletes and might require a higher level of performance to produce more reliable results. The test design was only applied to healthy subjects, and therefore caution must be taken when using results for injured athletes.

## 5. Conclusions

Soft and rigid knee braces do not affect performance in healthy participants. Missing experience with the devices might explain a few influences on feedback mechanisms. There is no disadvantage to be expected regarding healthy participants running back to sports test batteries with a brace or orthosis. Further studies after knee injuries are required.

## Figures and Tables

**Table 1:** Test protocol.

Studied Group	Group A (*n* = 8)	Group B (*n* = 8)	Group C (*n* = 8)
**Test run preceded by a warm-up**			
**Run 1**	No aid	Brace	Orthosis
**Run 2**	Brace	Orthosis	No aid
**Run 3**	Orthosis	No aid	Brace

*n*, number of individuals.

**Table 2:** Test statistics for between-group differences.

		SQUARE SUM	Chi^2^/F *	Sig.	Effect Size (ETA Square)
**Two-legged stability**	Friedmann		2.396	0.302	0.031
**One-legged stability**	Friedmann		1.542	0.463	0.026
**Plyometric jump test**	ANOVA		1.203	0.303	0.053
**Two-legged CMJ**	ANOVA		0.598	0.528	0.025
**one-leg CMJ**	ANOVA		0.536	0.589	0.023
**Parkour test**	ANOVA		0.643	0.484	0.027
**Quick feet test**	ANOVA		0.765	0.471	0.032

* F for ANOVA (Analysis of Variance repeated measures) repeated measures, Chi-Square for Friedman Test.

## Data Availability

Data is available from the authors on request from the corresponding author.
